# Single-Atom Fe Nanozyme with Enhanced Oxidase-like Activity for the Colorimetric Detection of Ascorbic Acid and Glutathione

**DOI:** 10.3390/bios13040487

**Published:** 2023-04-18

**Authors:** Yue Gu, Zhongxu Cao, Mengde Zhao, Yanan Xu, Na Lu

**Affiliations:** School of Materials Science and Engineering, Shanghai University of Engineering Science, Shanghai 201620, China

**Keywords:** single-atom nanozyme, Fe-N-C catalysts, oxidase-like, colorimetric detection, ascorbic acid, glutathione

## Abstract

Single-atom nanozymes (SAzymes) have drawn ever-increasing attention due to their maximum atom utilization efficiency and enhanced enzyme-like activity. Herein, a facile pyrolysis strategy is reported for the synthesis of the iron–nitrogen–carbon (Fe-N-C) SAzyme using ferrocene trapped within porous zeolitic imidazolate framework-8 (ZIF-8@Fc) as a precursor. The as-prepared Fe-N-C SAzyme exhibited exceptional oxidase-mimicking activity, catalytically oxidizing 3,3′,5,5′-tetramethylbenzidine (TMB) with high affinity (*K*_m_) and fast reaction rate (*V*_max_). Taking advantage of this property, we designed two colorimetric sensing assays based on different interaction modes between small molecules and Fe active sites. Firstly, utilizing the reduction activity of ascorbic acid (AA) toward oxidized TMB (TMBox), a colorimetric bioassay for AA detection was established, which exhibited a good linear range of detection from 0.1 to 2 μM and a detection limit as low as 0.1 μM. Additionally, based on the inhibition of nanozyme activity by the thiols of glutathione (GSH), a colorimetric biosensor for GSH detection was constructed, showing a linear response over a concentration range of 1–10 μM, with a detection limit of 1.3 μM. This work provides a promising strategy for rationally designing oxidase-like SAzymes and broadening their application in biosensing.

## 1. Introduction

Along with the rapid progress made in nanoscience and nanotechnology, various nanomaterials have been applied in many significant fields in biosensing [1,2,3,4,5], energy storage [6,7,8,9], and catalysis [10,11,12,13]. Over the past two decades, nanomaterials with enzyme-like characteristics, termed nanozyme, have attracted great attention because of their easy preparation, good stability in harsh conditions, and low cost. Up to now, a variety of nanomaterials have been discovered with enzyme-mimicking activity, such as transition metal oxide-based nanomaterials [14,15,16,17], noble metal- or metal-based nanostructures [18,19,20], carbon-based nanomaterials [21,22,23], and metal–organic framework-based nanostructures (MOFs) [24,25,26]. Although some progress has been made in this field, conventional nanozymes still face great challenges such as insufficient enzyme-like activity and low selectivity compared with natural enzymes.

Recently, single-atom nanozymes (SAzymes) have become a hot research topic [27]. As the atomically dispersed metal active sites of SAzymes are similar to the active centers of natural metalloenzymes, they have been expected to improve enzyme-like activities. Among them, Fe-N-C nanostructures have been supposed to be ideal nanozymes due to coordinated Fe-Nx active sites and abundant C-N moieties. Up to now, Fe-N-C nanozymes with different coordination structures have been developed and applied in various fields such as bioassays [28,29], tumor therapy [30,31], wound antibacterial applications [32], and organic pollutants degradation [33]. However, most of the reported Fe-N-C nanozymes are peroxidase mimics, and thus a novel approach to fabricate oxidase-like Fe-N-C nanozyme is required.

Here, we successfully prepared a Fe-N-C SAzyme with atomically dispersed Fe-Nx sites by pyrolysis of precursors consisting of ferrocene embedded in zeolitic imidazolate framework-8 (ZIF-8@Fc) under high temperature. The Fe-N-C SAzyme showed an exceptional oxidase-mimicking catalytic activity. Based on the different interaction modes between small molecules and Fe-N-C, two simple, sensitive, and selective colorimetric assays were established, which were used for the detection of ascorbic acid (AA) and glutathione (GSH).

## 2. Materials and Methods

### 2.1. Synthesis of Fe-N-C

Firstly, 1.19 g zinc nitrate hexahydrate was dissolved in 15 mL of mixed solvent consisting of methanol and *N*,*N*-dimethylformamide (DMF) (with a volume ratio of 4:1). Subsequently, the prepared solution was added to a 10 mL mixed solvent consisting of methanol and DMF (with a volume ratio of 4:1) containing 2.628 g of 2-methylimidazole until complete dissolution. Then, the above mixed solution was stirred for 3 min and left at room temperature for 10 h. Finally, the white precipitate was collected by centrifugation and washed with methanol for several times. With this, zeolitic imidazolate framework-8 (ZIF-8) was obtained.

The as-obtained ZIF-8 was dissolved in 40 mL of methanol. Then, 9 mg of ferrocene (Fc) was dissolved in 10 mL of methanol to obtain a homogeneous solution. Subsequently, the two solutions were mixed and stirred at room temperature for 4 h. The faint yellow precipitate of ZIF-8@Fc was obtained by centrifugation and washed with methanol for several times.

The above ZIF-8@Fc was transferred into a quartz boat and heated from room temperature to 900 °C at a heating rate of 3 °C min^−1^ under a nitrogen atmosphere placed in a tube furnace, followed by heating at 900 °C for 3 h. The pyrolyzed products were treated with 0.5 M H_2_SO_4_. Finally, the Fe-N-C catalysts were obtained after washing with methanol and drying in a vacuum oven.

### 2.2. Catalytic Activity of Fe-NC Nanozymes

Firstly, 6 μL of 1 mg mL^−1^ Fe-N-C was added to 291 μL of HAc-NaAc buffer (0.2 M, pH 4.0). Then, 3 μL of 10 mM 3,3′,5,5′-tetramethylbenzidine (TMB) was quickly added, and the mixed solution was incubated at room temperature for 10 min. Finally, absorption spectra were recorded by a UV–vis spectrometer.

A steady-state kinetic assay was performed at room temperature by adding different concentrations of TMB (10, 20, 30, 50, 80, 100, 200, and 300 μM).

### 2.3. Colorimetric Detection of AA

Firstly, 0.1 mM TMB was added to a HAc-NaAc buffer containing 20 μg mL^−1^ of Fe-N-C, and then the mixture was incubated for 10 min at room temperature. Subsequently, varying concentrations of AA (0, 0.1, 0.5, 1, 1.5, 2, 5, 10, 20, 40, 50, 80, and 100 μM) were introduced, followed by incubation for 1 min. Finally, the absorption spectra were recorded by a UV–vis spectrometer.

### 2.4. Colorimetric Detection of GSH

In brief, 20 μg mL^−1^ of Fe-N-C and varying concentrations of GSH (0, 1, 2, 5, 10, 20, 40, 50, 80, and 100 μM) were added to a HAc-NaAc buffer solution, and then 0.1 mM TMB was immediately added of. After incubation for 10 min at room temperature, the absorption spectra were recorded by a UV–vis spectrometer.

## 3. Results and Discussion

### 3.1. Synthesis and Characterization of Fe-N-C

**Scheme 1:**
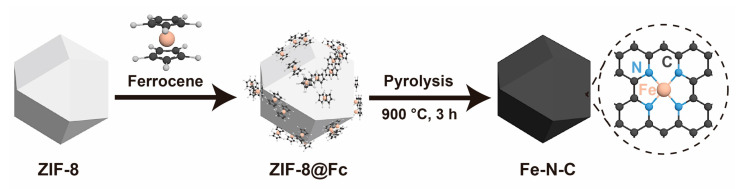
Schematic illustration of the synthesis process for Fe-N-C.

The Fe-N-C nanostructures were fabricated through a pyrolysis strategy. As illustrated in Scheme 1, ZIF-8 was first synthesized, followed by the trapping of Fc as a Fe source. Figures S2 and S3 revealed that the Brunauer–Emmett–Teller (BET) area of ZIF-8 was 1978.8 m^2^ g^−1^, and the average pore diameter was 14.2 Å. The unique pore structure of ZIF-8 allowed the successful adsorption of Fc and the atomically dispersion of Fe sites in ZIF-8@Fc. After a high-temperature pyrolysis treatment, ZIF-8 evolved into a carbon support, while zinc atoms evaporated, and the Fe atoms were chemically bonded with N atoms, resulting in the formation of Fe-Nx catalytic sites.

**Figure 1:**
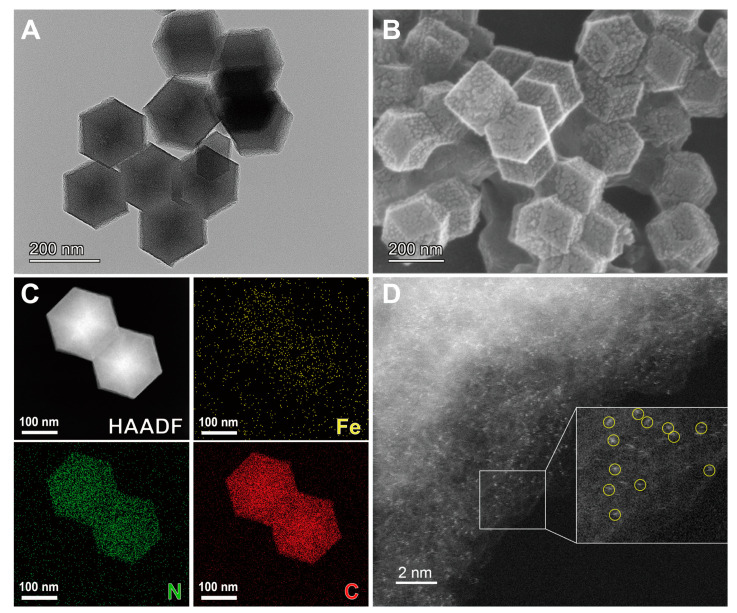
TEM (**A**) and SEM (**B**) images of Fe-N-C. (**C**) HAADF-STEM image and corresponding EDS elemental mapping of Fe, N, and C for the Fe-N-C catalyst. (**D**) AC-HAADF-STEM image of Fe-N-C.

Next, the morphology and structure of the as-prepared Fe-N-C were characterized. As shown in transmission electron microscope (TEM) and scanning electron microscope (SEM) images (Figure 1A,B), Fe-N-C retained the original dodecahedron shape of ZIF-8 (Figure S1) but showed some shrinkage and roughness on the surface. Further, high-angle circular dark field-scanning transmission electron microscopy images (HAADF-STEM) image and energy-dispersive spectroscopy (EDS) mapping (Figure 1C) showed the homogeneous distribution of Fe, N, and C elements in the whole nanoframework of Fe-N-C. Aberration-corrected high-angle annular dark-field scanning transmission electron microscopy (AC-HAADF-STEM) images clearly revealed isolated dispersed bright dots, highlighted by yellow circles (Figure 1D), which indicated the formation of Fe single atoms.

**Figure 2:**
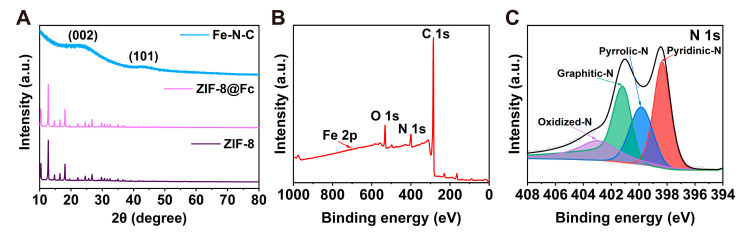
(**A**) XRD patterns of Fe-N-C, ZIF-8@Fc, and ZIF-8. (**B**) XPS survey spectrum of Fe-N-C. (**C**) XPS spectrum of N 1s for Fe-N-C.

Then, X-ray diffraction (XRD) was used to explore the graphitization degree of the pyrolyzed sample. As shown in Figure 2A, ZIF-8@Fc possessed similar characteristic diffraction peaks to those of ZIF-8, suggesting the trapping of Fc could not change the architecture of ZIF-8. After pyrolysis, the XRD patterns of Fe-N-C revealed two broad peaks located at 24° and 43°, corresponding to the (002) and (101) crystal faces of graphitic carbon, respectively. In the spectrum from X-ray photoelectron spectroscopy (XPS) (Figure 2B), we clearly observed the presence of C, N, Fe, and O elements in Fe-N-C, which was consistent with the results of EDS elemental mapping. The atomic fractions of Fe and N were 0.07 and 6.14%, respectively (Table S1). The high-resolution N 1s spectrum of Fe-N-C was deconvoluted into four fitted peaks at 398.36, 399.85, 401.19, and 402.97 eV (Figure 2C), which were assigned to pyridinic N, pyrrolic N, graphitic N, and oxidized N species, respectively [30]. Obviously, pyridinic and graphitic N were found to be the dominant N species, which served as anchor points for Fe atoms and contributed to enhance the enzyme-like activity of Fe-Nx sites. Nevertheless, the signal of Fe 2p in XPS could not be probed clearly (Figure S4), which might be ascribed to the low loading of Fe atoms on the sample surfaces.

### 3.2. Oxidase-like Activity of Fe-N-C

**Figure 3:**
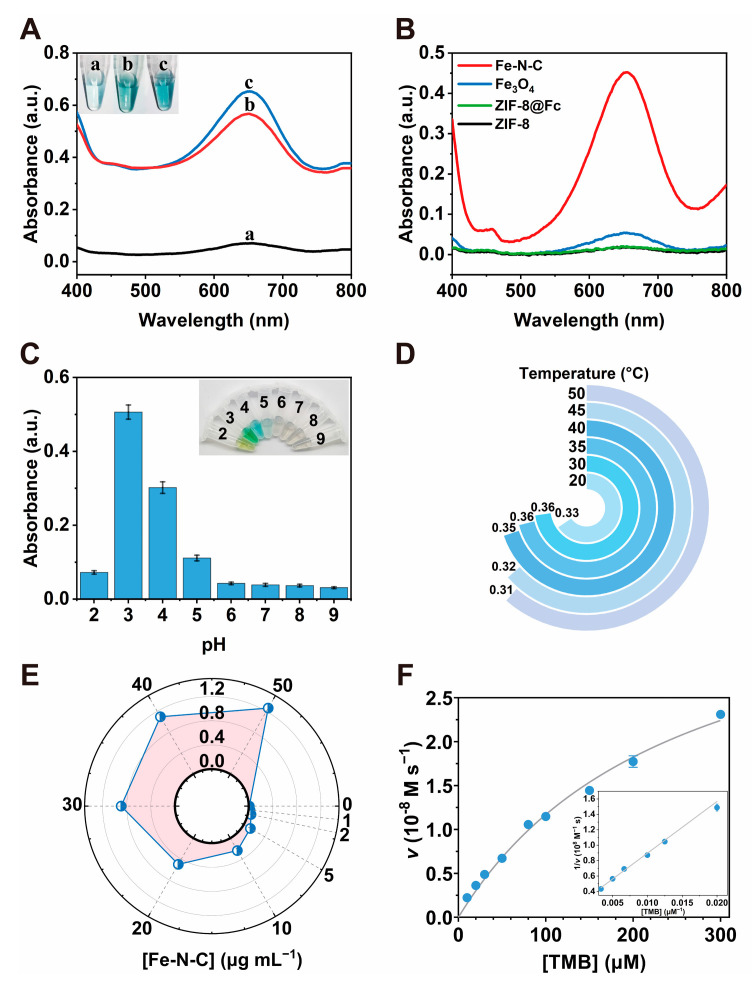
(**A**) UV–vis absorption spectra of (a) TMB + H_2_O_2_, (b) TMB + Fe-N-C, and (c) TMB + Fe-N-C + H_2_O_2_. Inset: corresponding photo of the color reaction. [TMB] = 0.1 mM; [H_2_O_2_] = 1 mM; [Fe-N-C] = 20 mg mL^−1^. (**B**) Comparison of the oxidase-like activity of different nanomaterials. (**C**–**E**) Effect of pH (**C**), temperature (**D**), and catalyst concentration (**E**) on the oxidase-like activity of Fe-N-C. (**F**) Steady-state kinetic curve of Fe-N-C toward TMB. Inset: Lineweaver–Burk plot.

The enzyme-like activity of Fe-N-C was evaluated by using TMB as a chromogenic substrate. In the presence of H_2_O_2_, the colorless TMB was catalytically oxidized, generating a blue oxidized TMB product (TMBox) with a characteristic absorption peak at 652 nm. As displayed in Figure 3A, when Fe-N-C was incubated with TMB for 10 min, we observed an obvious absorption peak at 652 nm, as well as an apparent color change from colorless to blue. In contrast, in the presence of Fe-N-C and H_2_O_2_, only a little higher absorbance at 652 nm was observed compared to that in normoxia. These results indicated the intrinsic oxidase-like activity of Fe-N-C. By comparison, it was found, as shown in Figure 3B, that Fe-N-C exhibited significantly enhanced oxidase-like activity compared with the ZIF-8@Fc precursor, the ZIF-8 precursor, and Fe_3_O_4_ nanoparticles (NPs).

Then, we explored the effect of pH, temperature, and catalyst concentration on the oxidase-like activity of Fe-N-C. As depicted in Figure 3C, the oxidase-like activity of Fe-N-C was dependent on the pH, providing a maximum absorbance at pH 3. To avoid the leaching of ferrous or ferric irons in acid conditions, a mild HAc-NaAc (pH 4) buffer was chosen as the reaction solution. Satisfactorily, Fe-N-C exhibited a similar catalytic performance in a range of temperatures (Figure 3D). For convenience, room temperature was employed in the subsequent assays. Moreover, the catalytic activity of Fe-N-C significantly increased with the increasing catalyst concentration (1–20 μg mL^−1^), further presenting a tendency of increasing slowly (Figure 3E). Thus, the optimal concentration of Fe-N-C was 20 μg mL^−1^. In addition, the reaction time curve (Figure S5) showed that 10 min was the optimal time.

To obtain kinetic parameters for Fe-N-C, a steady-state kinetic assay with TMB was conducted. As illustrated in Figure 3F, the reaction catalyzed by Fe-N-C demonstrated a typical Michaelis–Menten curve within a suitable TMB concentration range (0–300 μM). According to the fitted Lineweaver–Burk plot, the Michaelis constant (*K*_m_) and the reaction velocity (*V*_max_) were obtained. As reported in Table S2, the *K*_m_ value of Fe-N-C toward TMB was 48 times lower than that of glucose oxidase, suggesting a high affinity toward the substrate. As listed in Table S2, compared to other nanozymes, Fe-N-C exhibited favorable *K*_m_ and *V*_max_ values, which probably resulted from the uniform F-Nx active sites in Fe-N-C.

### 3.3. Colorimetric Assay for AA Based on the Reduction Activity

**Figure 4:**
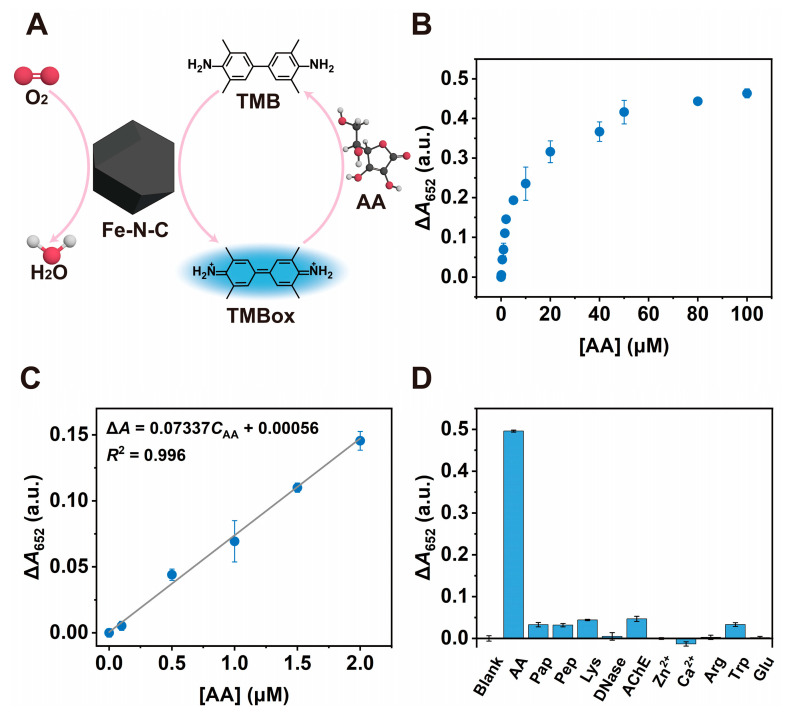
Colorimetric detection of AA based on reduction activity. (**A**) Schematic illustration of the AA-sensing principle. (**B**) Relationship between the relative absorbance at 652 nm (Δ*A*_652_) and the concentration of AA. (**C**) Linear calibration plot for AA detection. (**D**) Selectivity test. Concentrations of the interferents: 100 μM AA, 25 mM Zn^2+^ and Ca^2+^, and 10 mM Arg, Try, and Glu, 2.5 U mL^−1^ Pap, Pep, Lys, DNase, and AChE.

AA, an enzyme cofactor, plays an important role in many physiological and biochemical processes. An inappropriate AA content can result in many diseases, such as cancer, liver damage, and Alzheimer’s disease [34,35,36]. Moreover, AA is generally employed as an effective antioxidant in food, beverages, and pharmaceuticals, present in fruits and vegetables. Therefore, it is significant to establish a simple and efficient strategy for the determination of AA. Herein, based on the oxidase-mimicking activity of Fe-N-C and the strong reduction effect of AA on the TMBox, a simple and label-free colorimetric method for AA detection was established (Figure 4A). As displayed in Figure 4B, the relative absorbance at 652 nm (Δ*A*_652_) (Δ*A*_652_ = *A*_0_ − *A*, where *A*_0_ and *A* represent the absorbance at 652 nm in the absence and presence of AA, respectively) augmented gradually as the concentration of AA increased owing to the reduction activity of AA. Figure 4C revealed a good linear relationship between relative absorbance and AA concentration ranging from 0.1 to 2 μM, with a good linear regression equation, Δ*A* = 0.07337*C*_AA_ + 0.00056 (*R*^2^ = 0.996). According to the signal-to-noise ratio of 3σ rule, the limit of detection was calculated to be 0.1 μM. As reported in Table S3, the sensitivity of our proposed AA biosensor was higher than or comparable to those of the previously reported AA-sensing platforms.

To evaluate the selectivity of the AA-sensing platform, some possible interferents commonly present in human serum such as amino acids, biologically related metal ions, and enzymes were chosen for testing. These included tryptophan (Trp), arginine (Arg), glutamic acid (Glu), Zn^2+^, Ca^2+^, papain (Pap), pepsin (Pep), lysozyme (Lys), deoxyribonuclease (DNase), and acetylcholinesterase (AChE). As shown in Figure 4D, none of the interferents generated a significant relative absorbance change, even though their concentrations were 100, 250 times higher than that of AA or their activities were very high. This suggested that the colorimetric AA assay we designed possessed high selectivity toward the target analyte.

### 3.4. Colorimetric Assay for GSH Based on Its Inhibitory Effect

**Figure 5:**
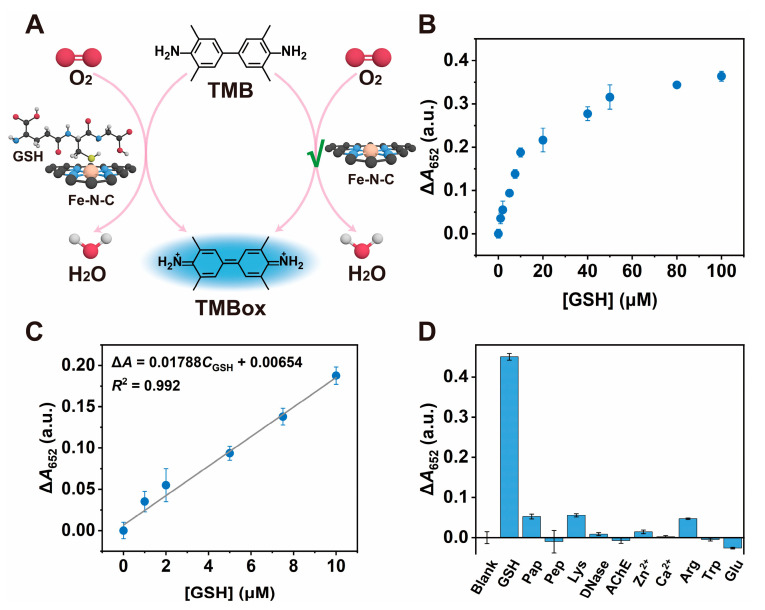
Colorimetric detection of GSH based on its inhibitory effect. (**A**) Schematic illustration of the GSH-sensing principle. (**B**) Relationship between the relative absorbance at 652 nm (Δ*A*_652_) and the concentration of GSH. (**C**) Linear calibration plot for GSH detection. (**D**) Selectivity test. Concentrations of the interferents: 100 μM GSH, 25 mM Zn^2+^ and Ca^2+^, 10 mM Arg, Try, and Glu, and 2.5 U mL^−1^ Pap, Pep, Lys, DNase, and AChE.

GSH is an endogenous thiol antioxidant in organisms. It serves essential roles in maintaining cellular redox homeostasis, radical signal transduction, and the regulation of immune system functions. An abnormal level of GSH in the body can lead to a variety of illnesses [37,38]. Hence, the development of a simple and sensitive method for GSH detection has become more and more crucial for disease diagnosis. It has been reported that thiols tend to coordinate with Fe atoms, resulting in the poisoning of Fe-based sites and preventing the interaction with oxygen, thereby reducing the catalytic activity of nanocatalysts. Based on the inhibitory effect of GSH on the oxidase-like activity of Fe-N-C, we developed a simple and effective colorimetric assay for GSH detection, shown in Figure 5A. As can be seen in Figure 5B, the relative absorbance at 652 nm was enhanced along with the increasing concentration of GSH, which was due to the strong inhibition of GSH of TMB oxidation. It was further found that the absorbance showed a linear relationship with the GSH concentration in the range of 1–10 μM (Figure 5C). The linear regression equation was Δ*A* = 0.01788*C*_GSH_ + 0.00654 (*R*^2^ = 0.992), and the limit of detection was 1.3 μM. By comparison with other reported GSH sensors, our proposed GSH biosensor demonstrated high sensitivity (Table S4).

In addition, interference experiments were performed in the presence of some possible interferents found in human serum. As displayed in Figure 5D, although the concentration of the interferents was 100, 250 times greater than that of GSH, their corresponding colorimetric signal changes were almost negligible, which suggested the excellent selectivity of our biosensor for GSH detection.

### 3.5. Practical Applications

To evaluate the application of Fe-N-C in practical tests, the contents of AA and GSH spiked in human serum samples were determined. As shown in Tables S5 and S6, the recovery of AA and GSH ranged from 96.1% to 111.9% and from 95.7% to 102.7%, respectively. In addition, the RSD were less than 6.3% and 3.0%, respectively. Hence, we established reliable sensing platforms for AA and GSH detection, which demonstrated great potential in real applications.

## 4. Conclusions

In summary, a facile high-temperature pyrolysis strategy was utilized to prepare a single-atom Fe-N-C nanozyme using Fc molecules trapped within ZIF-8 (ZIF-8@Fc) as precursors. The as-prepared Fe-N-C showed enhanced oxidase-like activity, which could be ascribed to the uniform Fe-Nx active sites. As proof-of-concept applications, we utilized the Fe-N-C nanozyme to develop a sensing platform for AA detection based on AA reduction activity, as well as another sensing platform for GSH detection based on GSH inhibitory effect. The proposed Fe-N-C-based colorimetric strategies for AA and GSH analysis showed high sensitivity and good selectivity. Our proposed biosensors possess several merits. First, the construction of such biosensors only requires one nanomaterial and is very simple and easy to operate. Secondly, the proposed biosensors present excellent analytical performances, and their sensitivities were better than or comparable to those of previously reported sensing platforms. Thirdly, the proposed colorimetric assays are label-free, rapid (~10 min), and cost-effective, which are beneficial features for point-of-care testing (POCT). They provide a promising analytical method for the detection of biological small molecules.

## Data Availability

The data are available under request to the correspondence.

## Supplementary Materials

The following supporting information can be downloaded at: https://www.mdpi.com/article/10.3390/bios13040487/s1, Experimental section: Chemicals and Materials, Apparatus.; Figure S1: TEM (A) and SEM (B) images of ZIF-8; Figure S2: N_2_ adsorption/desorption isotherms of ZIF-8; Figure S3: BET plot with linear regression for ZIF-8; Figure S4: XPS spectrum for Fe 2p; Figure S5: Effect of the reaction time on the oxidase-like activity of Fe-N-C; Table S1: Atomic percentage of Fe-N-C; Table S2: Comparison of kinetic parameters for Fe-N-C and other oxidase mimics [39,40,41,42,43,44,45]; Table S3: Comparison of kinetic parameters for Fe-N-C and other oxidase mimics [46,47,48,49,50,51,52,53]; Table S4: Comparison of the sensing performance of our proposed method and other reported GSH sensors [54,55,56,57,58,59,60]; Table S5: Results of the determination of AA in normal human serum samples; Table S6: Results of the determination of GSH in normal human serum samples.
