# Supplementary material for: Single-Atom Fe Nanozyme with Enhanced Oxidase-like Activity for the Colorimetric Detection of Ascorbic Acid and Glutathione
Source: Biosensors (Basel). 2023 Apr 18;13(4):487. doi: 10.3390/bios13040487 (PMC10137000; doi:10.3390/bios13040487)
Supplement: Supplementary file 1 [file biosensors-13-00487-s001.zip › biosensors-2220674-supplementary.pdf]

Table of Contents

EXPERIMENTAL SECTION..S3
Figure S1. TEM (A) and SEM (B) images of ZIF-8..............................S4
Figure S2. N_2 adsoption/desorption isotherms of ZIF-8. ....S5
Figure S3. BET-Plot with linear regression for ZIF-8............................S6
Figure S4. XPS spectrum for Fe 2p...S7
Figure S5. Effect of reaction time on oxidase-like activity of Fe-N-C...S8
Table S1. Atomic percentage of the Fe-N-C...S9
Table S2. Comparison of kinetic parameters for Fe-N-C and other oxidase mimics. S10
Table S3. Comparison of the sensing performance of our proposed method with other reported AA sensors...S11
Table S4. Comparison of the sensing performance of our proposed method with other reported GSH sensors. S12
Table S5. Results for the determination of AA in normal human serum samples. S13
Table S6. Results for the determination of GSH in normal human serum samples. S14
References S15

EXPERIMENTAL SECTION

Chemicals and Materials. 3,3',5,5'-tetramethylbenzidine (TMB), ascorbic acid (AA), glutathione (GSH), tryptophan (Trp), arginine (Arg), and glutamic acid (Glu) were obtained from Sigma-Aldrich (Shanghai, China). Papain (Pap), pepsin (Pep), lysozyme (Lys), deoxyribonuclease (DNase), and acetylcholinesterase (AChE) were obtained from Beyotime Biotechnology (Shanghai, China). Ferrocene (Fc), and zinc nitrate hexahydrate ($Zn(NO_3)_2$, 99.5%), were purchased from Alfa Aesar (Shanghai, China). 2-Methylimidazole (98%), 30% hydrogen peroxide (H_2O_2), acetic acid (HAc), sodium acetate (NaAc), and sulfuric acid (H_2SO_4) (98.08%) were purchased from Adamas-beta (Shanghai, China). Methanol (99.5%) and ethanol (99.7%) were purchased from General-reagent (Shanghai, China). Ultrapure water was used in the experiments involving aqueous solution. All chemicals were used without further purification. Ultrapure water with a resistivity of 18 $M\Omega \cdot cm^{-1}$ was used in the experiments.

Apparatus. UV-vis spectra were recorded by using UV-2600 spectrometer (Shimadzu, Japan). The pore parameters such as Brunauer-Emmett-Teller (BET) and Barrett-Joyner-Halenda (BJH) pore size distribution were measured using N2 adsorption/desorption at 393 K on a Quantachrome Autosorb IQ3 (USA). Transmission electron microscopy (TEM) image and high-angle annular dark-field scanning transmission electron microscopy (HAADF-STEM) image were obtained on Talos F200S G2 transmission electron microscope (Thermo Fisher Scientific, America). Scanning electron microscopy (SEM) image was photographed by TESCAN MIRA3 LM field emission scanning electron microscope (TESCAN, Czech). Powder X-ray diffraction (XRD) patterns were measured by X'Pert3 Powder X-ray diffractometer (PANalytical, Netherlands). X-ray Photoelectron Spectroscopy (XPS) spectra were obtained by K-Alpha X-ray photoelectron spectrometer (Thermo Fisher Scientific, America).

Pretreatment of serum samples. Firstly, 0.3 μL serum was incubated with 0.2 μL N-Ethylmaleimide NEM (100 mM) for 2 h at room temperature. Then, centrifugated the mixed solution for 10 min. Finally, transferred the supernatant as pretreated serum to prepare for the next experiment.

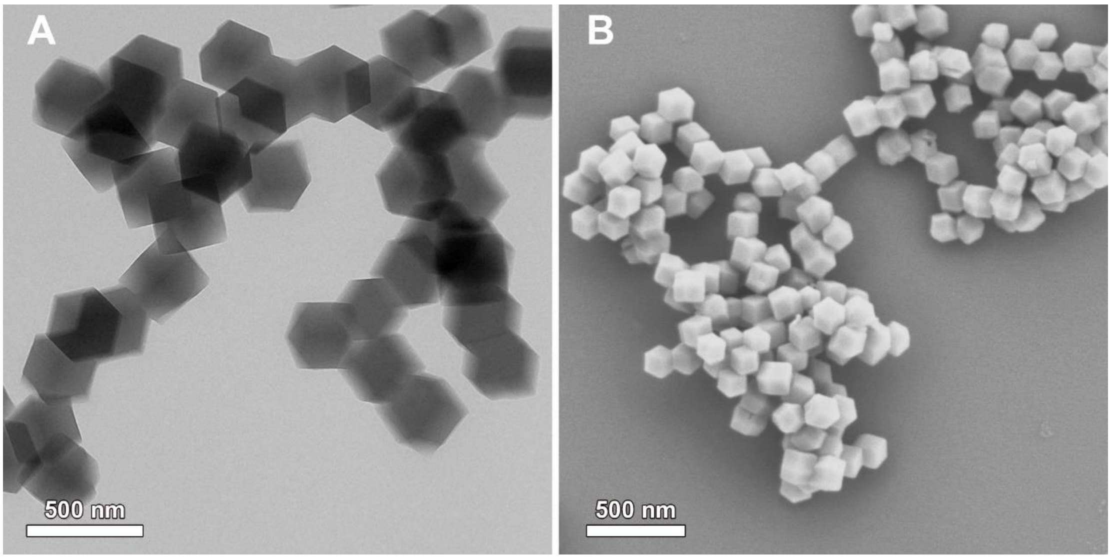

Figure S1. TEM (A) and SEM (B) images of ZIF-8.

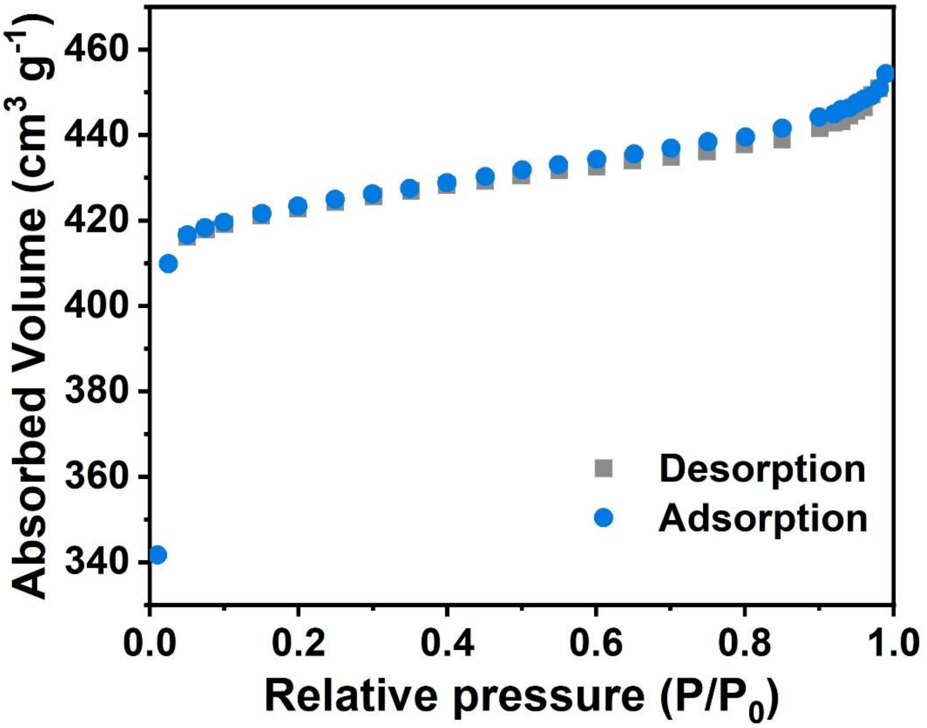

Figure S2. N_2 adsorption/desorption isotherms of ZIF-8.

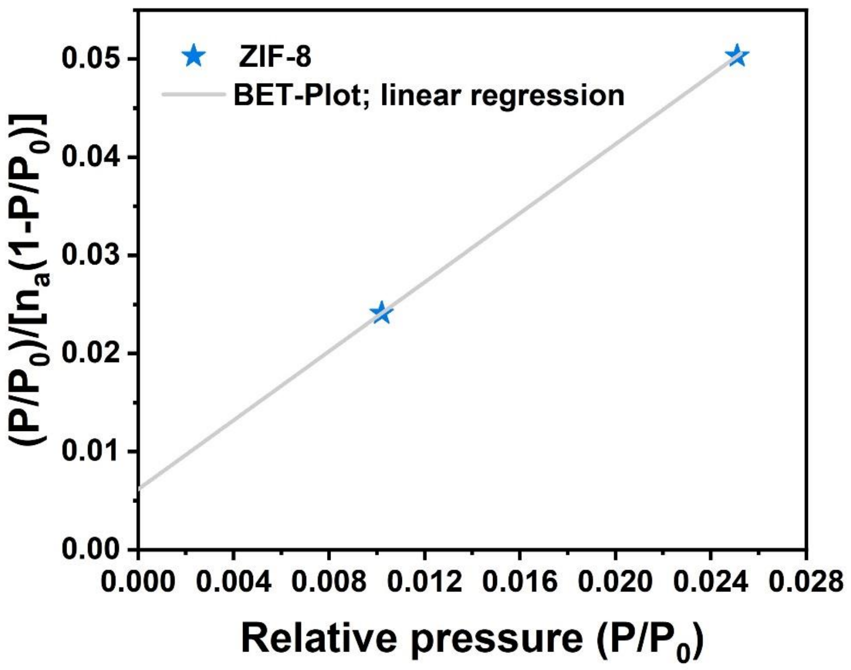

Figure S3. BET-Plot with linear regression for ZIF-8.

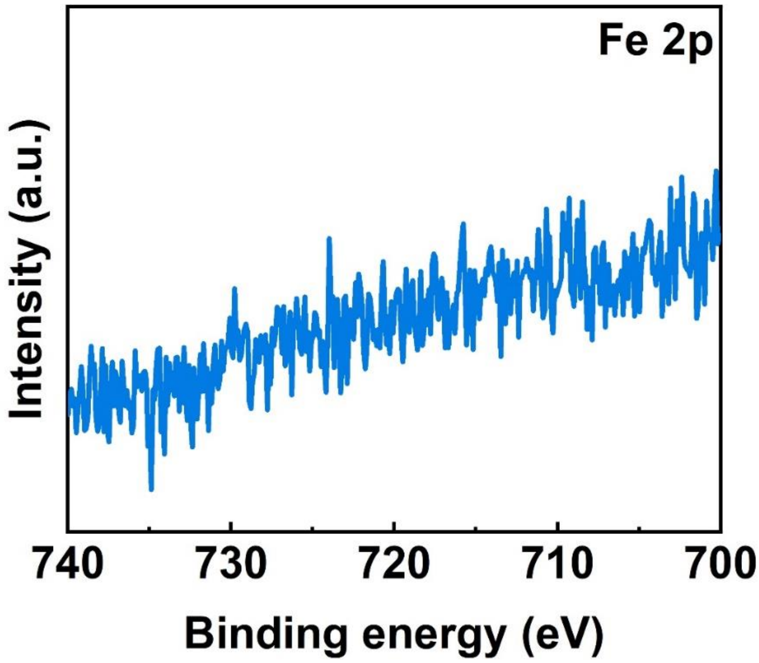

Figure S4. XPS spectrum for Fe 2p.

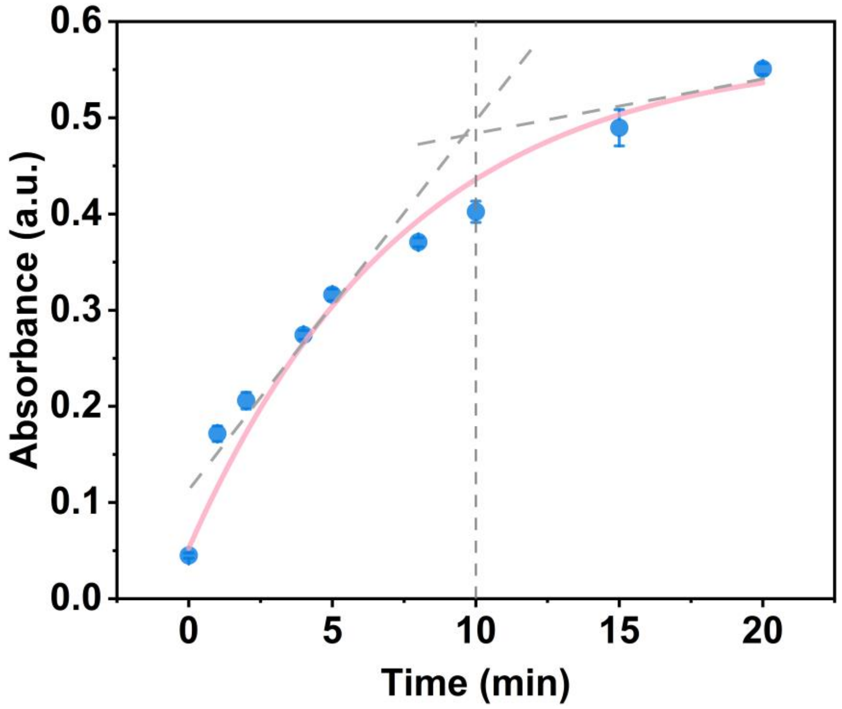

Figure S5. Effect of reaction time on oxidase-like activity of Fe-N-C.

Table S1. Atomic percentage of the Fe-N-C.

Sample	Fe 2p (%)	N 1s (%)	C 1s (%)	O 1s (%)
Fe-N-C	0.07	7.23	86.45	6.25

Table S2. Comparison of kinetic parameters for Fe-N-C and other oxidase mimics.

Catalyst	K_m (mM)	V_{max} (10^{-8} M s^{-1})	Ref.
[a] Fe-N/C-CNT (Fe-N_3)	0.13	2.25	[1]
[b] Fe-N-C SAC	1.81	0.601	[2]
[c] Fe-N-C-400	0.269	33.80	[3]
Co-SAC	0.578	34.5	[4]
Ni-N/C-CNTs	0.284	0.245	[5]
Fe-N/C-CNTs	0.62	52.6	
Co-N/C-CNTs	1.17	20.8	
FeBi-NC	0.21	0.7	[6]
Glucose oxidase	12.1	60.1	[7]
Fe-N-C	0.253	4.136	This work

[a] Fe-N/C-CNT (Fe-N_3): ferrum-nitrogen-carbon carbon nanotube (with Fe-N_3 units);

[b] Fe-N-C SAC: Fe-N-C single-atom catalyst;

[c] Fe-N-C-400: Fe-N-C under carbonization at 400 °C.

Table S3. Comparison of the sensing performance of our proposed method with other reported AA sensors.

Nanomaterial	Method	Linear range	LOD	Ref
CuO/Pt	Colorimetry	1 μM−0.6 mM	0.796 μM	[8]
MOF-808	Colorimetry	30 μM−1 mM	15 μM	[9]
[a] CL/TiN/GCE	Electrochemistry	50−1500 μM	1.52 μM	[10]
[b] ITO/rGO/AuNPs	Electrochemistry	20−150 μM	9.4 μM	[11]
[c] PPy@C/GCE	Electrochemistry	10−150 μM	0.75 μM	[12]
Cu-Ag/rGO nanocomposite	Colorimetry	5−30 μM	3.6 μM	[13]
[d] PPYNTs/Co_3O_4	Electrochemistry	5−80 μM	0.23 μM	[14]
[e] PMo_{12}-PANI/β-CD/ITO	Electrochemistry	0.01−3000 μM	0.015 μM	[15]
Fe-N-C	Colorimetry	0.1−2 μM	0.123 μM	This work

[a] CL/TiN/GCE: glassy carbon electrode modified with chrysanthemum-like titanium nitride nanostructures;

[b] ITO/rGO/AuNPs: indium tin oxide-based electrodes with both reduced graphene oxide and Au nanoparticles;

[c] PPy@C/GCE: glassy carbon electrode modified with polypyrrole grafted cellulose;

[d] PPYNTs/Co_3O_4: polypyrrole nanotubes with Co_3O_4 nano powder;

[e] PMo_{12}-PANI/β-CD/ITO: polyaniline (PANI) and phosphomolybdic acid (PMo_{12}) co-polymerized on the film of β-cyclodextrin (β-CD) modified ITO.

Table S4. Comparison of the sensing performance of our proposed method with other reported GSH sensors.

Nanomaterial	Method	Linear range	LOD	Ref
Gold nanoclusters	Colorimetry	2−25 μM	0.42 μM	[16]
[a] COF-300-AR	Colorimetry	1−15 μM	1.0 μM	[17]
[b] N, S-CQDs	Fluorescence	0−100 μM	6.7 μM	[18]
[c] MnO_2-induced PDA-NPs	Fluorescence	0−350 μM	1.5 μM	[19]
[d] PDI-C_4SH	Electrochemistry	50−5000 μM	17 μM	[20]
[e] Receptor 1	Colorimetry	60−120 μM	5.86 μM	[21]
[f] Rho-GSH	Fluorescence	0−5000 μM	49.6 μM	[22]
Fe-N-C	Colorimetry	1−10 μM	1.31 μM	This work

[a] COF-300-AR: reductive product of COF-300;

[b] N, S-CQDs: nitrogen and sulfur co-doped carbon quantum dots;

[c] MnO_2-induced PDA-NPs: MnO_2-induced polydopamine nanoparticles;

[d] PDI-C_4SH: *N*, *N*''-2-octyloxy-1-methyl-2-oxoethyl)-(-2,4-thiolbutyloxy)-1-methyl-2-oxoethyl) -3,4:9,10-perylenetetracarboxyldiimide;

[e] Receptor 1: (E)-5-(diethylamino)-2-(((2-(methylthio) phenyl)imino)methyl)phenol;

[f] Rho-GSH: 3’, 6’-bis(ethylamino)-2’, 7’-dimethyl-2-(4-nitrop henoxy) spiro [isoindoline-1, 9′ -xanthen]-3-one.

Table S5. Results for the determination of AA in normal human serum samples.

Samples	AA spiked (μM)	Total detected (μM)	Recovery (%)	RSD (%, n = 3)
Serum 1	0.5	0.54	108.4	5.3
	1	1.10	109.6	5.4
	1.5	1.44	96.1	6.3
Serum 2	0.5	0.49	97.5	1.6
	1	1.12	111.9	0.7
	1.5	1.38	97.9	0.6
Serum 3	0.5	0.61	110.0	0.4
	1	1.11	111.5	1.9
	1.5	1.49	99.1	1.1

Table S6. Results for the determination of GSH in normal human serum samples.

Samples	GSH spiked (μM)	Total detected (μM)	Recovery (%)	RSD (%, n = 3)
Serum 1	0	1.23	-	0.8
	2	3.18	98.4	1.5
	4	5.11	97.8	1.9
	8	9.29	100.7	2.5
Serum 2	0	1.25	-	1.4
	2	3.14	96.7	2.3
	4	5.02	95.7	0.8
	8	9.41	101.7	2.9
Serum 3	0	1.55	-	0.7
	2	3.64	102.7	1.9
	4	5.54	99.8	0.2
	8	9.49	99.4	1.3

References

1. Wang, Y.; Zhang, Z.; Jia, G.; Zheng, L.; Zhao, J.; Cui, X. Elucidating the mechanism of the structure-dependent enzymatic activity of Fe-N/C oxidase mimics. *Chem. Commun.* **2019**, *55*, 5271–5274.
2. Wu, Y.; Jiao, L.; Luo, X.; Xu, W.; Wei, X.; Wang, H.; Yan, H.; Gu, W.; Xu, B.Z.; Du, D.; et al. Oxidase-like Fe-N-C single-atom nanozymes for the detection of acetylcholinesterase activity. *Small* **2019**, *15*, e1903108.
3. Xu, Y.; Xue, J.; Zhou, Q.; Zheng, Y.; Chen, X.; Liu, S.; Shen, Y.; Zhang, Y. The Fe-N-C nanozyme with both accelerated and inhibited biocatalytic activities capable of accessing drug-drug interactions. *Angew. Chem.* **2020**, *59*, 14498–14503.
4. Liu, X.; Ding, H.; Hu, B.; Tian, F.; Sun, J.; Jin, L.; Yang, R. Large-scale synthesis of high loading Co single-atom catalyst with efficient oxidase-like activity for the colorimetric detection of acid phosphatase. *Appl. Surf. Sci.* **2022**, 605, 154766.
5. Zhao, C.; Xiong, C.; Liu, X.; Qiao, M.; Li, Z.; Yuan, T.; Wang, J.; Qu, Y.; Wang, X.; Zhou, F.; et al. Unraveling the enzyme-like activity of heterogeneous single atom catalyst. *Chem. Commun.* **2019**, *55*, 2285–2288.
6. Chen, Q.; Liu, Y.; Lu, Y.; Hou, Y.; Zhang, X.; Shi, W.; Huang, Y. Atomically dispersed Fe/Bi dual active sites single-atom nanozymes for cascade catalysis and peroxymonosulfate activation to degrade dyes. *J. Hazard. Mater.* **2022**, *422*, 126929.
7. Avan, A.N.; Demirci-Cekic, S.; Apak, R. Colorimetric nanobiosensor design for determining oxidase enzyme substrates in food and biological samples. *ACS Omega* **2022**, *7*, 44372–44382.
8. Wang, X.; Han, Q.; Cai, S.; Wang, T.; Qi, C.; Yang, R.; Wang, C. Excellent peroxidase mimicking property of CuO/Pt nanocomposites and their application as an ascorbic acid sensor. *Analyst* **2017**, *142*, 2500–2506.
9. Zheng, H.Q.; Liu, C.Y.; Zeng, X.Y.; Chen, J.; Lu, J.; Lin, R.G.; Cao, R.; Lin, Z.J.; Su, J.W. MOF-808: A metal-organic framework with intrinsic peroxidase-like catalytic activity at neutral pH for colorimetric biosensing. *Inorg. Chem.* **2018**, *57*, 9096–9104.
10. Zhang, L.; Feng, J.; Chou, K.-C.; Su, L.; Hou, X. Simultaneously electrochemical detection of uric acid and ascorbic acid using glassy carbon electrode modified with chrysanthemum-like titanium nitride. *J. Electroanal. Chem.* **2017**, *803*, 11–18.
11. Mazzara, F.; Patella, B.; Aiello, G.; O'Riordan, A.; Torino, C.; Vilasi, A.; Inguanta, R. Electrochemical detection of uric acid and ascorbic acid using r-GO/NPs based sensors. *Electrochim. Acta* **2021**, *388*, 138652.
12. Shalini, A.; Paulraj, P.; Pandian, K.; Anbalagan, G.; Jaisankar, V. Single pot synthesis, characterization of PPy@C composites modified electrode for the electrocatalytic determination of ascorbic acid in commercial fruit samples. *Surf. Interfaces* **2019**, *17*, 100386.
13. Darabdhara, G.; Sharma, B.; Das, M.R.; Boukherroub, R.; Szunerits, S. Cu-Ag bimetallic nanoparticles on reduced graphene oxide nanosheets as peroxidase mimic for glucose and ascorbic acid detection. *Sens. Actuators B Chem.* **2017**, *238*, 842–851.
14. Ganesha, H.; Veeresh, S.; Nagaraju, Y.S.; Suresh, D.S.; Devendrappa, H. Micelles self-degraded polypyrrole nanotube-cobalt oxide nanocomposite based electrochemical sensor for detection of Ascorbic acid. *Inorg. Chem. Commun*. **2022**, *145*, 109975.
15. Yu, X.; Zhou, Q.; Bi, L. Ultrasensitive electrochemical sensor based on β-cyclodextrin–polyaniline–phosphomolybdic acid matrix for the detection of ascorbic acid. Russ. *J. Appl. Chem*. **2023**, *95*, 1036–1047.
16. Feng, J.; Huang, P.; Shi, S.; Deng, K.Y.; Wu, F.Y. Colorimetric detection of glutathione in cells based on peroxidase-like activity of gold nanoclusters: A promising powerful tool for identifying cancer cells. *Anal. Chim. Acta* **2017**, *967*, 64–69.
17. Jin, P.; Niu, X.; Zhang, F.; Dong, K.; Dai, H.; Zhang, H.; Wang, W.; Chen, H.; Chen, X. Stable and reusable light-responsive reduced covalent organic framework (COF-300-AR) as a oxidase-mimicking catalyst for GSH detection in cell lysate. *ACS Appl. Mater. Interfaces* **2020**, *12*, 20414–20422.
18. Song, Z.; Quan, F.; Xu, Y.; Liu, M.; Cui, L.; Liu, J. Multifunctional N,S co-doped carbon quantum dots with pH- and thermo-dependent switchable fluorescent properties and highly selective detection of glutathione. *Carbon* **2016**, *104*, 169–178.
19. Kong, X.J.; Wu, S.; Chen, T.T.; Yu, R.Q.; Chu, X. MnO_2-induced synthesis of fluorescent polydopamine nanoparticles for reduced glutathione sensing in human whole blood. *Nanoscale* **2016**, *8*, 15604–15610.
20. Perk, B.; Büyüksünetçi, Y.T.; Hakli, Ö.; Xue, C.; Li, Q.; Anik, Ü. Centri-voltammetric GSH detection with PDI-CSH as a carrier material. *ChemistrySelect* **2021**, *6*, 11648–11652.
21. You, G.R.; Jang, H.J.; Jo, T.G.; Kim, C. A novel displacement-type colorimetric chemosensor for the detection of Cu^{2+} and GSH in aqueous solution. *RSC Adv.* **2016**, *6*, 74400–74408.
22. Zhou, Z.; Li, P.; Liu, Z.; Wu, C.; Zhang, Y.; Li, H. Construction of a unique fluorescent probe for rapid and highly sensitive detection of glutathione in living cells and zebrafish. *Talanta* **2022**, *243*, 123364.
